# Comparison of the Oocyte Quality Derived from Two-Dimensional Follicle Culture Methods and Developmental Competence of* In Vitro* Grown and Matured Oocytes

**DOI:** 10.1155/2018/7907092

**Published:** 2018-04-04

**Authors:** Jaewang Lee, Eun Jung Kim, Hyun Sun Kong, Hye Won Youm, Seul Ki Kim, Jung Ryeol Lee, Chang Suk Suh, Seok Hyun Kim

**Affiliations:** ^1^Department of Biomedical Laboratory Science, Eulji University, Seongnam, Gyeonggi-do, Republic of Korea; ^2^Department of Obstetrics and Gynecology, Seoul National University Bundang Hospital, Seongnam, Gyeonggi-do, Republic of Korea; ^3^Department of Obstetrics and Gynecology, Seoul National University College of Medicine, Seoul, Republic of Korea; ^4^Department of Obstetrics and Gynecology, Seoul National University Hospital, Seoul, Republic of Korea

## Abstract

*In vitro* follicle growth (IVFG) is an emerging fertility preservation technique, which can obtain fertilizable oocytes from an* in vitro* culture system in female. This study aimed to compare efficiency of the most widely used two-dimensional follicle culture methods [with or without oil layer (O+ or O− group)]. Preantral follicles were isolated from mice and randomly assigned. Follicles were cultured for 10 days and cumulus-oocyte complexes harvested 16–18 hours after hCG treatment. Follicle and oocyte growth, hormones in spent medium, meiotic spindle localization, expression of reactive oxygen species (ROS), mitochondrial activity, and gene expression were evaluated. In follicle growth, survival, pseudoantral cavity formation, ovulation, and oocyte maturation were also significantly higher in O+ group than O− group. Hormone production was significantly higher in follicles cultured in O+ than O−. There were no significant differences in mRNA expression related to development. On the other hand, the level of ROS was increased while the mitochondrial activity of* in vitro grown* matured oocyte was less than* in vivo* matured oocytes. In conclusion, follicle culture with O+ group appears to be superior to the culture in O− group in terms of follicle growth, development, oocyte growth, maturation, and microorganelles in oocyte.

## 1. Background

Given that cancer diagnosis and treatment have dramatically improved in recent decades, fertility preservation for cancer survivors has become more important. In female cancer patients, embryo and oocyte cryopreservation are the most clinically used for fertility preservation. However, these methods have several disadvantages, including potential delays in cancer treatment, the need for hormone injections during stimulation, and availability of an appropriate sperm donor [1–3]. Most importantly, these options are not available for prepubertal girls [1, 4]. Ovarian tissue (OT) cryopreservation and transplantation methods could constitute alternative options, with >60 babies documented as being born as a result of this method [5].

However, OT cryopreservation and transplantation carry potential risks of hematological malignant cell reimplantation after OT transplantation, because cancer cells can still reside in cryopreserved OTs [6–9]. However,* in vitro *follicle growth and maturation from OTs and acquisition of developmentally competent oocytes can avoid such concerns [2, 4, 10]. Therefore, this technique provides an alternative option for fertility preservation without the risk of cancer cell reimplantation following OT transplantation. Since the first report by Eppig and Schroeder [11], diverse culture methods, such as the absence or presence of an oil layer under 2D culture, group culture, and three-dimensional follicle culture, have been used in follicle culture systems [12–15]. Specifically, 2D culture systems have been primarily applied to harvest* in vitro*-grown and matured oocytes in mice [16–18], rats [19], bovines [20], monkeys [21], and humans [22]. However, few studies have compared 2D culture systems [23].

Although follicle culture systems are useful for mature oocyte collection, maturational and developmental competence of* in vitro*-grown oocytes decrease, compared with* in vivo*-grown and -matured oocytes [16]. Much effort has expanded to improve the quality of* in vitro*-grown oocytes, while the main cause of their decreased quality remains unknown. Here, we compared different 2D culture systems to determine the mechanisms associated with impaired oocyte quality during ovarian-follicle culture in mice.

## 2. Materials and Methods

### 2.1. Animals and Ovarian Tissue Collection

Female BDF-1 mice were obtained from Orient Bio (Seongnam, Korea). The experimental protocols and animal-handling procedures were performed with approval of the Institutional Animal Care and Use Committee (IACUC) of Seoul National University Bundang Hospital (BA1506-178/028-01). All procedures were performed using IACUC-approved methods. Following sacrifice of the mice by cervical dislocation, ovaries were collected in collection medium Dulbecco's phosphate buffered saline (D-PBS; Biowest, L0615-500, Nuaille, France) supplemented with 5% heat-inactivated fetal bovine serum (FBS; Gibco, Carlsbad, 10438-026, CA, USA).

### 2.2. Isolation and Allocation of Secondary Follicles

Ovaries were aseptically dissected from 2-week-old BDF-1 female mice (*n* = 90) and then mechanically isolated using fine-needles. Early secondary follicles (diameter 110–130 *μ*m) were randomly divided into two different groups according to the culture methods (presence or absence of oil overlay) as previous researches [12, 14, 23]. A total of 2,880 mouse ovarian follicles were used to compare the efficiency of the 2D culture methods for secondary ovarian follicles as previous study. Follicles with intact basal membrane showing no gaps between the oocyte and surrounding granulosa cells were regarded as healthy and then selectively collected by observation under microscope [12, 13, 23].

### 2.3. Culture Medium and Conditions (Oil Overlay versus without Oil Layer)

The culture medium used for follicles was *α*-minimum essential medium Glutamax (*α*-MEM; Life technologies, 32571-036, Carlsbad, CA, USA) containing 5% FBS, 5 *μ*g/mL insulin, 5 *μ*g/mL transferrin, 5 ng/mL sodium selenite (Sigma-Aldrich, St. Louis, I3146, MO, USA), 1% penicillin-streptomycin (PS, 15140-122, Sigma), and 10 mIU/mL of recombinant human follicle stimulating hormone (rhFSH; Gonal-F; Merck Serono, GONAL-F 450, Geneva, Switzerland).

Follicles were individually plated onto two different culture plates according to the culture system as two previous studies [12, 13]. First, the follicles were cultured under a mineral oil overlay (O+ group) to minimize osmotic change similar to a previous study [13]. Briefly, culture dishes (60-mm Petri dishes; Becton Dickinson, Franklin Lakes, NJ, USA) contained 16 drops of 20 *μ*L medium and were covered with 5 mL of mineral oil (Sigma-Aldrich, M5310). Selected follicles were individually seeded in the culture droplets. Half of the culture medium (10 *μ*L) was changed every other day. The second culture system was established according to a previous study [12]. Briefly, selected follicles were allocated at one follicle/well in 96-well plates (SPL, Pocheon, Korea) containing 75 *μ*L/well culture medium without mineral oil overlay (O− group). Every 4 days, 30 *μ*L of the culture medium was replenished to maintain the culture environment. Follicles were grown in an incubator at 37°C, 100% humidity, and 5% CO_2_ atmosphere. Follicles obtained from both culture systems were cultured* in vitro *for 10 days. To assess the growth of individual follicles, two perpendicular diameters of follicles were measured every other day using a calibrated ocular micrometer at 50x magnification, with viable follicles defined as those that retained an oocyte completely embedded within the granulosa cell mass [12]. Survival rate was calculated as a percentage of all plated follicles as described previously [13]. Additionally, antral-like cavity formation was defined as visible lucent space in the granulosa cell complex around the oocyte and was evaluated at the end of culture day 10 [12].

### 2.4. Oocyte Maturation and Classification

On the 10th day of culture,* in vitro*-grown follicles were treated with 1.5 IU/mL human chorionic gonadotropin (hCG; CG5, Sigma-Aldrich) and 5 ng/mL murine epidermal growth factor (EGF; E4127, Sigma-Aldrich) regardless of their size to induce oocyte maturation at the germinal vesicle (GV) stage. After 16–18 h, mucified cumulus-oocyte complexes (COCs) were selected and oocytes were denuded with 85 IU/mL hyaluronidase (Sigma-Aldrich) to assess oocyte maturational status. Oocytes were individually classified into GV, GV breakdown (GVBD), and metaphase II (MII) stages according to the presence of GVs or first polar body extrusion. For controls, mature oocytes were obtained from 4-week-old BDF-1 mice superovulated with 5 IU pregnant mare serum gonadotropin, followed by injection of 5 IU hCG 48 h later. Oocytes were collected from the oviduct 14–16 h after hCG injection, and cumulus cells were removed by treatment with 85 IU/mL hyaluronidase as described previously [24].

### 2.5. Enzyme-Linked Immunosorbent Assay (ELISA) for Estradiol (E2) and Progesterone (P4)

The concentrations of E2 (D4, D8, and D10) and P4 (D8, D10, and D11) in spent medium were determined by ELISA (E2: Calbiotech, Spring Valley, CA, USA; and P4: DRG diagnostics, Springfield, NJ, USA). Absorbance was measured at 450 nm, and the concentrations of E2 and P4 were calculated against corresponding concentrations of E2 (pg/mL) and P4 (ng/mL) from a standard curve.

### 2.6. Immunofluorescence to Evaluate Spindle Normality

To localize the nucleus, cortical granule (CGs), microfilaments, and meiotic spindles, each oocyte was immediately fixed with 4% paraformaldehyde (Sigma-Aldrich, 158127) for 30 min after denudation. Oocytes were then washed with 0.3% BSA (Sigma-Aldrich, A3311) twice and permeabilized with 0.1% Triton X-100 in D-PBS for 15 min. Eggs were blocked in PBS containing 3% BSA at 4°C overnight, and then the oocytes were incubated with rabbit anti-*β*-tubulin antibody (1 : 100; Cell Signaling, 2128S, Danvers, MA, USA) for 1 h at room temperature (RT). After incubation, oocytes were randomly divided into two groups according to the staining of microorganelles, CGs, or microfilaments. To visualize CGs or microfilaments, oocytes were stained with rhodamine-conjugated* Lens culinaris* agglutinin (1 : 200; Vector Laboratories, RL-1042, Burlingame, CA, USA) or rhodamine-conjugated phalloidin (1 : 100; Molecular Probes, R415, Eugene, OR, USA), respectively. Alexa Fluor 488-conjugated secondary goat anti-rabbit IgG (1 : 1,000; Thermo Fisher Scientific, A11008, Waltham, MA, USA) and Hoechst 33342 (Sigma-Aldrich, B2261) were added at RT for a 1-h incubation to visualize meiotic spindles and nuclei, respectively. Oocytes with barrel-shaped bipolar spindles and well-organized microtubule fibers, along with tightly aligned chromosomes on the metaphase plate, were scored as normal. All other configurations were considered abnormal [25].

### 2.7. Evaluation of Reactive Oxygen Species (ROS) Production and Mitochondrial Activity in Oocytes

ROS production and mitochondrial activity were detected and measured with fluorescence staining.* In vitro*- and* in vivo*-matured oocytes were separately incubated with 200 nM of ROS-detection reagents (Molecular Probes, C6827), 100 nM of Mitotracker mitochondrion-selective probes (Molecular Probes, M7512), and Hoechst 33342 at 37°C for 30 min. Positive controls were treated with 1 mM hydrogen dioxide for 30 min to induce artificial oxygenic stress, and the probes were omitted from negative controls. Images were detected and captured on a LSM 710 confocal microscope (Carl Zeiss, Oberkochen, Germany), and fluorescence intensity was measured using Zen 2012 software (Carl Zeiss) and presented as arbitrary units (AUs).

### 2.8. Gene Expression in Oocytes

To evaluate oocyte maturational ability, cell death, and developmental competence, gene expression was determined by quantitative reverse-transcription PCR (qRT-PCR). On the 11th day of the culture period, mature oocytes were collected 14–16 h after hCG treatment, and mRNAs were extracted and pooled from five oocytes in each group using Dynabeads mRNA direct micro kit (Dynal, Oslo, Norway) according to manufacturer instructions. cDNA was then synthesized using PrimeScript first-strand cDNA synthesis kit (Takara Bio, 6100B, Shiga, Japan), and qRT-PCR was performed using the following primer sets listed as Table 1. The threshold cycle (Ct) value represents the cycle number indicating increase in fluorescence above background levels. Reactions were performed according to protocols included with the SYBR premix dimer eraser PCR kit (Takara Bio, RR820L). The PCR protocol used a denaturation step of 95°C for 10 min, followed by an amplification and quantification program that was repeated 40 times (95°C for 15 s and 60°C for 1 min) and a melting curve program (60–95°C with a heating rate of 0.34/s and continuous fluorescence measurement). Gene expression in each group was analyzed by generating a melting curve. The size of the PCR products was confirmed by gel electrophoresis on 3% agarose gels stained with Loading Star (Intronbio, Seongnam, Korea) and visualized by ultraviolet light using ethidium bromide staining as described previously [26]. The relative quantification of gene expression was analyzed using the 2^−ΔΔCt^ method. For all qRT-PCR experiments, *β*-actin mRNA served as an internal standard in analyzed oocytes, and gene expression was normalized to* in vivo*-derived MII oocytes for comparison.

### 2.9. Statistical Analysis

Data were analyzed using the Chi-square test (for evaluation of oocyte nuclear maturity, follicle development, oocyte diameter, and spindle normality) or analysis of variance (for follicle diameter, hormone levels, ROS and mitochondrial activity, and gene expression). The Statistical Package for the Social Sciences version 12.0 software (SPSS Inc., Chicago, IL, USA) and GraphPad Prism 6.0 (GraphPad Software, La Jolla, CA, USA) were used for statistical analysis. Values were considered significant at *p* < 0.05.

## 3. Results

### 3.1. Follicle Survival, Growth, and Development

**Figure 1:**
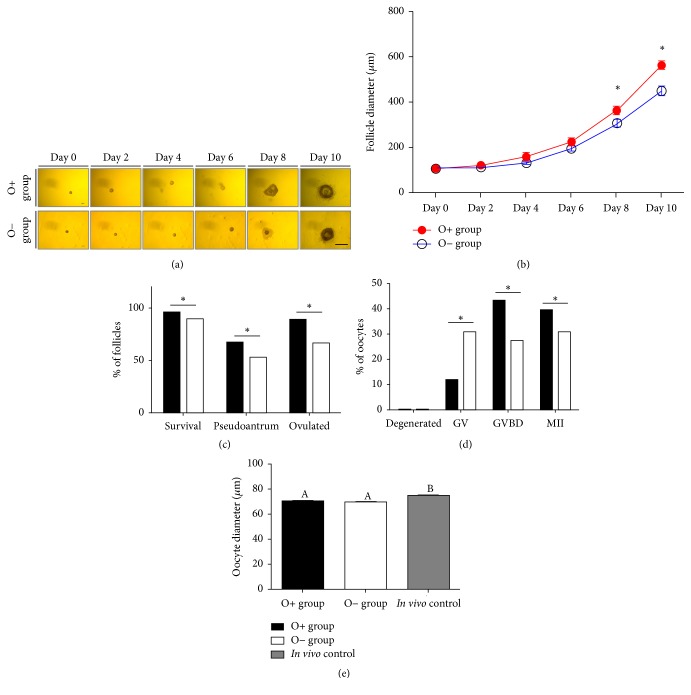
*Follicle growth and development and oocyte growth and development according to different 2D culture methods*. ((a) and (b)) Representative image of follicle growth and curve for follicle diameter during the culture period. (c) Follicular development criteria on the 10th day of culture. ((d) and (e)) Developmental stages and oocyte diameter. Asterisks and letters denote statistical difference (*p* < 0.05).

Figure 1(a) shows Morphological changes of ovarian follicles during the entire culture period.  Figure 1(b) shows the growth curve of follicles from different culture milieu according to the culture period. From the 8th day of culture, follicle diameter in the O+ group was significantly larger, compared with that of follicles in the O− group (diameter on day 8: 364.7 ± 18.0 *μ*m versus 307.6 ± 14.6 *μ*m; diameter on day 10: 566.0 ± 16.9 *μ*m versus 449.4 ± 16.8 *μ*m; *p* < 0.05).  Figure 1(c) represents survival, development, and ovulation rates (cumulus-oocyte complex formation) on the 10th day of culture. All of these criteria in the O+ group were also significantly higher than those observed in the O− group (survival: 95.8% versus 90.1%; pseudoantral-like cavity formation: 66.9% versus 52.6%; and ovulation rate: 89.1% versus 66.4%; *p* < 0.05).

### 3.2. Oocyte Growth and Maturation

On the 10th day of the culture period, hCG and EGF were administered for 14–16 h to induce oocyte maturation* in vitro*. Following hCG treatment, GVBD- and MII formation rates in the O+ group increased significantly, compared with those measured in the O− group, whereas the percentage of GV oocytes decreased significantly (*p* < 0.05; Figure 1(d)). After COC denudation, the oocyte diameters in both* in vitro* culture systems (O+ and O− groups) were significantly smaller than those observed in* in vivo*-derived oocytes (O+ group: 69.8 ± 0.5 *μ*m; O− group: 69.6 ± 0.5 *μ*m.;* in vivo* control: 74.8 ± 0.4 *μ*m.; *p* < 0.05; Figure 1(e)).

### 3.3. E2 and P4 Production in Spent Medium

**Figure 2:**
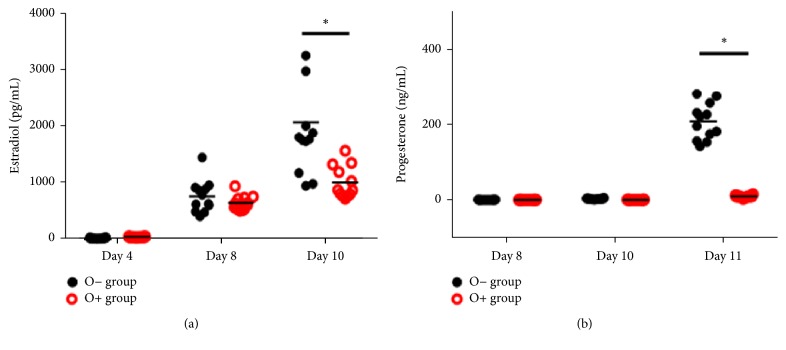
*Hormone production during the culture period in accordance with the culture method*. ((a) and (b)) Estradiol and progesterone levels in spent culture medium at each evaluation day of the culture period. Each plot indicates each hormone value, with asterisks denoting statistical difference (*p* < 0.05).

With respect to E2 levels in spent medium, we observed no significant difference between culture systems until the 8th day of culture. However, E2 levels in the O− group were significantly higher than those measured in the O+ group on the 10th day of culture (Figure 2(a); *p* < 0.05).

In contrast, no significant difference was observed in P4 levels until the end of the follicle culture (8th and 10th days of culture). Following oocyte maturation, both* in vitro *culture systems exhibited increased P4 levels in spent medium, compared with levels observed at the end of the culture (day 10). P4 levels in the O− group were significantly higher than that of the O+ group on the 11th day of culture (Figure 2(b); *p* < 0.05).

### 3.4. Oocyte Microorganelles

**Figure 3:**
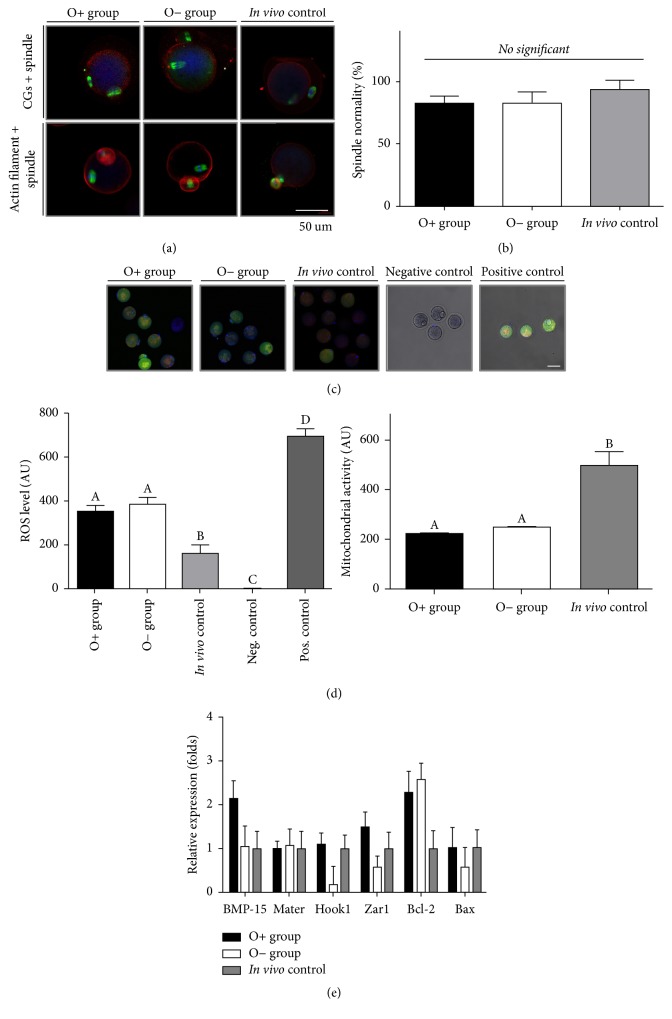
*Quality of cytoplasmic and nuclear maturation according to the 2D culture method*. (a) Distribution of cortical granules, actin filaments, and meiotic spindles of* in vitro*- and* in vivo-*derived oocytes. (b) Normal spindle organization. (c) Reactive oxygen species (ROS) production and mitochondrial activity in different groups. (d) Graphs showing quantitative fluorescent intensity illustrating ROS production and mitochondrial activity. Different letters indicate statistically significant differences among groups. (e) mRNA expression in oocytes derived from three different* milieu*.

Figure 3(a) shows CGs, microfilaments, and meiotic spindles in oocytes. CGs in oocytes derived from both* in vitro* culture methods were clumped and not evenly distributed; however, CGs of* in vivo*-matured oocytes were evenly distributed at the marginal side of the oocyte. By contrast, oocytes from all three groups were similar in terms of actin-filament localization. However, there were no significant differences in spindle normality among the three groups (O+ group: 81.0 ± 3.6%; O− group: 82.4 ± 4.5;* in vivo* control: 92.9 ± 4.1%; Figure 3(b)).

### 3.5. Intracellular ROS Levels and Mitochondrial Activity

Figure 3(c) shows representative images of ROS production and mitochondrial activity in* in vitro*- and* in vivo*-matured oocytes. When measuring oxygenic stress in oocytes from all three groups, oocytes derived from* in vivo* conditions exhibited significantly lower levels than those of both* in vitro*-grown and matured oocytes (O+ group: 350.4 ± 29.3 AU; O− group: 384.7 ± 31.9 AU;* in vivo *control: 161.5 ± 39.6 AU; negative control: 1.9 ± 0.15 AU; positive control: 694.4 ± 34.4 AU; *p* < 0.05; Figure 3(d)). Mitochondrial activity in oocytes from* in vivo* milieu was significantly increased, compared with the activities measured in both groups of* in vitro*-derived oocytes (O+ group: 225.3 ± 9.7 AU; O− group: 246.9 ± 11.1 AU;* in vivo *control: 496.7 ± 55.2 AU; *p* < 0.05; Figure 3(d)).

### 3.6. Gene Expression

We performed qRT-PCR to determine the cause of impairment in the developmental competence of oocytes derived from* in vitro *culture conditions. Our results indicated no statistical differences in expression levels between various genes related to oocyte growth, maturation, embryonic development, and cell death among the three different groups (Figure 3(e)).

## 4. Discussion

Due to the risk of reimplanting malignant cells following OT transplantation,* in vitro* follicle culture was developed to provide an alternative instead of OT cryopreservation and transplantation for fertility preservation in female cancer patients [22]. Although several studies attempted to establish* in vitro* follicle culture systems, only few studies involving mouse models have achieved success in the form of live-birth deliveries [11, 15, 27]. Conventional cultures (2D) with or without an oil layer for individual follicle cultures and other methods, such as multiple-follicle cultures, have been applied in most studies [11, 27–29]. Recently, three-dimensional cultures using the extracellular matrix were adopted to mimic three-dimensional structures for follicle growth and maturation [15, 30]. Despite these efforts, multiple issues remain concerning maturational and developmental errors associated with follicle culture systems.

In this study, we applied two different 2D follicle culture systems for individual secondary follicle cultures in order to compare the efficiency of 2D culture methods. Although there are different 2D culture systems for multiple-follicle culturing, we did not use these, because multiple-follicle cultures are unable to trace individual follicle growth maturation. Additionally, these methods would not allow for accurate measurement of follicle diameter or ruling out paracrine effects from multiple-follicle culture systems. Therefore, we compared only two different 2D culture methods for individual cultures. In this study, we cultured secondary follicle not primordial/primary follicle because individual* in vitro *primary follicle has some limitations including cell death, growth retardation, maturational failure, and others [31, 32]. Moreover, only few studies have reported the success of primordial follicle* in vitro* culture in nonhuman primate [33] and human [34–36]. However, they did not obtain mature oocyte from primordial follicle culture* in vitro.* It means that an* in vitro* primordial follicle culture method is not still established completely.

With respect to follicle growth, development, oocyte maturation, and somatic cell proliferation and function, the culture conditions of the O+ group appeared slightly superior to those of the O− group. There were three differences between the O+ and O− groups. The first involved the presence or absence of an oil layer on the culture medium. We hypothesized that the oil layer prevented severe pH changes and osmolality during the handling and culture period [37], which can positively affect follicular growth, maturation, and oocyte nuclear maturation, given that oocytes are very sensitive to external changes, such as pH, osmolality, and temperature [38–43]. On the contrast to our expectation, the level of osmolality of two different culture medium was not significantly different (Supplementary Figure 1). Although we measured the osmolality of spent medium in the present study, we could not evaluate their pH and other characteristics. Thus, there still is possibility of difference in microenvironment of culture medium. This difference of microenvironment could be one of the reasons of the different follicle growth in the two different culture methods. Second, the volume of the culture medium (20 *μ*L) in the O+ group was less than that of the O− group (75 *μ*L). Because the aim of this study was to compare two widely used 2D follicle culture methods which used different media volumes, we hypothesized that smaller volumes of culture media for single-follicle cultures would constitute a more favorable environment in terms of autocrine effects. Ovarian follicles and oocytes exhibit autocrine effects during folliculogenesis and oocyte development, respectively. Secretomes under autocrine modulation may exist in higher concentrations in smaller volumes of medium, compared with larger volumes, and this may also influence follicle and oocyte development. Finally, the volume of replenishment medium was also different between the O+ and O− groups. Fifty percent of the culture medium (10 *μ*L) in the O+ group was exchanged every other day, whereas 40% of the culture medium in the 96-well (30 *μ*L) plates was refreshed every 4 days according to previous studies [12, 13]. To compare the hormone level in different amount of medium appropriately, we recalculated the hormone concentration by adjustment with different dilution factors (dilution factors for O+: 2 and O−:7.5, respectively). We hypothesized that larger volumes of changed media might play roles in the depletion of metabolic waste products, such as nitric oxide, ammonium, and glutamate, or in the maintenance of nutrients and pH to support follicle culture* in vitro *[44]. Unlike our expectation, the ovarian hormone (estradiol and progesterone) in O+ group was less than that of O− at the end of culture period. According to the previous study, because the presence of oil layer may have a sequestration effect on steroids, the level of steroid hormones we measured was decreased in O+ group [23].

We suggest that these three differences are possible causes of the significant differences observed in follicle growth and development. However, the two different* in vitro *culture systems did not show differences in terms of characteristics or quality of mature oocytes, although follicle growth and development were different. Based on measurements involving follicle diameter, pseudoantral-like cavity formation, and hormone production, we propose that an* in vitro *culture condition might also influence somatic cell physiology and function, especially granulosa cells, and not directly affect oocyte development.

Although embryonic development and healthy live births were reported in mouse models, the efficiency was low [11, 13, 15, 27]. Obata et al. [45], using the same strain of mice used in this study (BDF-1), showed impairment of developmental competence in oocytes grown and matured* in vitro*. They overcame the impaired developmental competence by nuclear transfer, followed by IVF, and concluded that an* in vitro* follicle culture and denudation of cumulus cells caused critical cytoplasmic deficiency. Therefore, we suggest that decreased fertilization ability and embryonic development might be related to cytoplasmic defects and not nuclear defects. We separately analyzed nuclear and cytoplasmic status to determine the cause of developmental failure. Nuclear maturation was assessed by the proportion of first polar body extrusion (MII formation) and gene expression related to oocyte growth and maturation* (BMP-15)*, configuring the microtubule cytoskeleton, and regulating chromosome segregation* (Hook1)*, zygotic arrest* (Zar-1 and Mater)*, and apoptosis (*Bcl-2* and* Bax*) [46–53]. As we expected, we observed no significant difference in nuclear maturation.

In terms of cytoplasmic deficiency, we checked CG distribution in oocytes according to a previous report [24]. Oocytes from* in vitro* culture systems showed abnormal CG distribution in the form of clumping CGs, compared with* in vivo*-derived oocytes, which was consistent with our findings. CGs play a role in inducing the alteration of structure in zona pellucida to block polyspermy. Based on this finding, CG biogenesis and localization would be impaired in* in vitro*-derived oocytes.

We also investigated physiological characteristics including ROS production and mitochondrial expression. In both* in vitro*-derived oocyte groups, ROS production was significantly higher than that observed in* in vivo*-derived oocytes, whereas mitochondrial activity significantly decreased, compared with that measured in* in vivo* controls. Ovarian follicles should be exposed to high levels of oxygen (~20%) during the* in vitro* culture period, and they would have also experienced oxygenic stress from the* in vitro* culture milieu. Therefore, we suggest that massive production of ROS might cause decreased mitochondrial activity and negatively influence preimplantation embryonic development. According to a previous studies, mitochondrial activity is correlated with two-cell blocks, and the use of mitochondrial nutrients, such as coenzyme Q10, could improve the outcome of infertility treatment in older patients [54, 55].

According to previous studies, cytoplasmic replacement following nuclear transfer can overcome developmental failure and restoration of meiotic maturation and spindle assembly [45, 56]. It implies that the nucleus still possesses adequate potential for maturation and embryonic development. Our results also showed normal nuclear development and gene expression in both* in vitro*-derived oocytes. These findings indicated that developmental failure in oocytes derived from the* in vitro *milieu might be caused by cytoplasmic deficiency.

This study had several limitations. Although maintaining a three-dimensional microenvironment is thought to be crucial factor for human clinical trial, we only compared two culture methods for 2D follicle culture conditions; therefore, we did not evaluate the three-dimensional structure of follicles. Because three-dimensional follicle cultures have been widely used relatively recently, further study concerning three-dimensional follicle cultures should be undertaken. The most important aspect to consider prior to clinical application is species-specific differences between mice and humans. Moreover, we did not clarify the exact mechanisms regarding how the different culture methods effected folliculogenesis and oocyte maturation.

## 5. Conclusions

Here, we compared two different 2D follicle culture systems using a mouse model and* in vitro* follicle culture systems. Our results indicated that culture systems with an oil overlay appeared superior to other culture methods not using mineral oil. Additionally, we showed that the cytoplasmic deficiency of* in vitro*-derived oocytes might be a cause of reduced fertilization ability and embryonic development. Further studies on the optimization of culture conditions to improve the efficacy of follicle cultures and embryonic development are required.

## Figures and Tables

**Table 1:** Primer sequences for qRT-PCR.

Gene symbol	Primer sequence (5′ → 3′)	Reference	Genbank accession
Bax (F)	AGCGAGTGTCTGAAGCG	Varnosfaderani S. R. et al., (2013)	NM_007527.3
Bax (R)	CCCAGTTGAAGTTGCCGT		
Bcl2 (F)	CCTTCTTTGAGTTCGGAG	Varnosfaderani S. R. et al., (2013)	NM_009741.3
Bcl2 (R)	CCTTCAGAGACAGCCAG		
BMP-15 (F)	CAGTAAGGCCTCCCAGAGGT	Sanchez F. et al., (2009)	NM_009757.3
BMP-15 (R)	AAGTTGATGGCGGTAAACCA		
Hook1 (F)	GGCAGATACACTAGCATTTGA	Habibi A. et al., (2010)	NM_030014
Hook1 (R)	CTCCTCATTCGTCTCCTTCAG		
Mater (F)	CAATGCCCTGTCTCTAACCTG	Sanchez F. et al., (2009)	NM_011860.2
Mater (R)	TGTCTTCTCACTCGGGCATA		
Zar1 (F)	CTCAGGACCCCGGTGATT	Sanchez F. et al., (2009)	NM_174877.2
Zar1 (R)	CCGTACTTCTGCTCTAAGAACTGG		
*β*-Actin (F)	CCATCGGCAATGAGCGGT	Varnosfaderani S. R. et al., (2013)	NM_031144.2
*β*-Actin (R)	CGTGTTGGCGTAGAGGTC		

## Data Availability

There are no shared data and material for this manuscript.

## Abbreviations

IVFG: * In vitro* follicle growth
FP: Fertility preservation
OT: Ovarian tissue
OTCP: Ovarian tissue cryopreservation and transplantation
2D: Two-dimensional
3D: Three-dimensional
FBS: Fetal bovine serum
ITS: Insulin-transferrin-selenium
FSH: Follicle stimulating hormone
hCG: Human chorionic gonadotropin
EGF: Epidermal growth factor
COCs: Cumulus-oocyte complexes
GVBD: Germinal vesicle breakdown
MII: Metaphase II
E2: 17ß-estradiol
P4: Progesterone
ELISA: Enzyme-linked immunosorbent assay
CGs: Cortical granules
BSA: Bovine serum albumin
Ct: Threshold cycle
BMP-15: Bone morphogenetic protein-15
Mater: A maternal effect gene required for early embryonic development in mice
Hook1: Hook microtubule tethering protein 1
Zar1: Zygotic arrest 1
Bcl-2: B-cell lymphoma-2
Bax: Bcl-2 associated X protein
O+: Follicle culture with mineral oil
O−: Follicle culture without mineral oil.

## Supplementary Materials

Supplementary Materials Supplementary Figure  1 shows the osmolality change of spent medium during culture period in both O+ group and O- group. However, the osmolality in O- was comparable to that of O+ group during an* in vitro *follicle growth and even after oocyte maturation.
