# Supplementary figures and images for: Comparison of the Oocyte Quality Derived from Two-Dimensional Follicle Culture Methods and Developmental Competence of In Vitro Grown and Matured Oocytes
Source: Biomed Res Int. 2018 Apr 4;2018:7907092. doi: 10.1155/2018/7907092 (PMC5904821; doi:10.1155/2018/7907092)

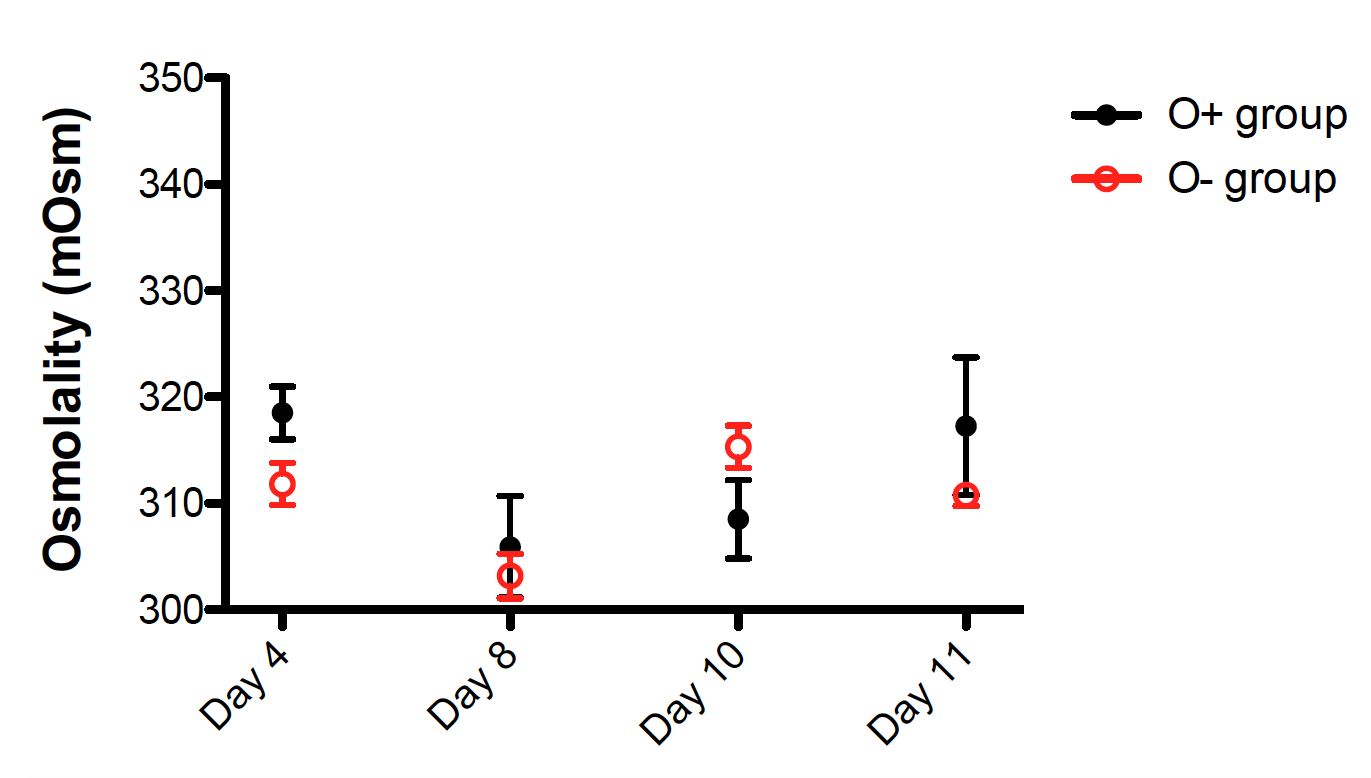

Supplement: Supplementary Materials — Supplementary Figure 1 shows the osmolality change of spent medium during culture period in both O+ group and O- group. However, the osmolality in O- was comparable to that of O+ group during an in vitro follicle growth and even after oocyte maturation. [file 7907092.f1.png]
